# YAP/TAZ-mediated regulation of laminin 332 is enabled by β4 integrin repression of ZEB1 to promote ferroptosis resistance

**DOI:** 10.1016/j.jbc.2024.107202

**Published:** 2024-03-18

**Authors:** Hira Lal Goel, Emmet R. Karner, Ayush Kumar, Dimpi Mukhopadhyay, Shivam Goel, Arthur M. Mercurio

**Affiliations:** Department of Molecular, Cell and Cancer Biology, University of Massachusetts Chan Medical School, Worcester, Massachusetts, USA

**Keywords:** Hippo, laminin, integrin, ZEB1, ferroptosis, differentiation

## Abstract

We are interested in the contribution of integrins and the extracellular matrix to epithelial differentiation in carcinomas. This study was motivated by our finding that the Hippo effectors YAP and TAZ can sustain the expression of laminin 332 (LM332), the predominant ECM ligand for the integrin β4, in breast carcinoma cells with epithelial differentiation. More specifically, we observed that YAP and TAZ regulate the transcription of the *LAMC2* subunit of LM332. Given that the β4–LM332 axis is associated with epithelial differentiation and YAP/TAZ have been implicated in carcinoma de-differentiation, we sought to resolve this paradox. Here, we observed that the β4 integrin sustains the expression of miR-200s that target the transcription factor ZEB1 and that ZEB1 has a pivotal role in determining the nature of YAP/TAZ-mediated transcription. In the presence of β4, ZEB1 expression is repressed enabling YAP/TAZ/TEAD-mediated transcription of *LAMC2*. The absence of β4, however, induces ZEB1, and ZEB1 binds to the *LAMC2* promoter to inhibit *LAMC2* transcription. YAP/TAZ-mediated regulation of LAMC2 has important functional consequences because we provide evidence that LM332 enables carcinoma cells to resist ferroptosis in concert with the β4 integrin.

The Hippo effectors YAP and TAZ are integral to the biology of epithelial cells, and they contribute to the initiation and progression of carcinomas (1). Among the many studies in this area, the seminal study that implicated TAZ in the biology of more de-differentiated breast cancers and the function of breast cancer stem cells (CSCs) (2) captured our attention because of our interest in understanding mechanisms that influence tumor differentiation and the function of CSCs. More specifically, we have had a long-standing interest in the role of integrins, especially the α6β4 integrin, in these processes, for example, (3, 4, 5, 6). The integrin α6β4, referred to as ‘β4 integrin,’ is an adhesion receptor for the laminins (LMs) (3), especially LM332, and it was characterized initially as an epithelial specific integrin (7, 8). Indeed, numerous studies have associated β4 with normal epithelia differentiation, for example (9), as well as with the function of carcinoma cells that retain epithelial characteristics (10, 11). In this context, we reported previously that exogenous expression of β4 in de-differentiated, transformed mammary epithelial cells is sufficient to promote their differentiation (12).

The current study was motivated by our goal of understanding better the relationship between YAP/TAZ and the β4 integrin in the context of breast carcinoma differentiation. In pursuit of this goal, we made the unexpected observation that YAP/TAZ can sustain the expression of LM332, the predominant matrix ligand for β4. Given that the β4–LM332 axis is associated with epithelial differentiation (9, 13), this observation raised the paradox of how YAP/TAZ can contribute to the expression of factors involved in epithelial differentiation and also promote de-differentiation. The results obtained revealed that β4 itself has a pivotal role in influencing the transcriptional activity of YAP/TAZ as exemplified by the ability of this integrin to enable YAP/TAZ-mediated transcription of the laminin C2 subunit (*LAMC2*), which is specific to LM332. In the absence of this integrin in more de-differentiated carcinoma cells, however, YAP/TAZ is unable to activate *LAMC2* transcription.

## Results

### TAZ-mediated regulation of LAMC2 is dependent on expression of the β4 integrin

**Figure 1:**
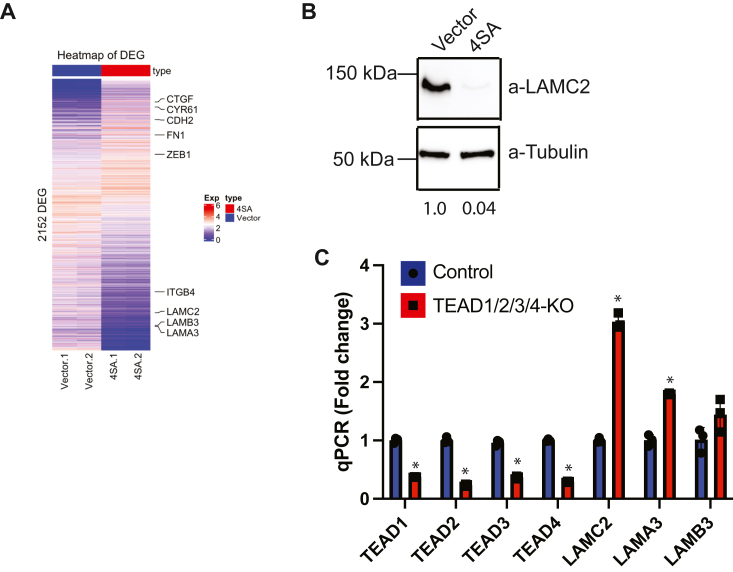
**LAMC2 is a direct target of TAZ.***A*, heatmap of RNA seq data comparing control and TAZ^4SA^-transformed S1 mammary epithelial cells. *B*, protein lysates from control and TAZ^4SA^-transformed S1 cells were immunoblotted using Abs specific for LAMC2 and tubulin. The immunoblot shown is representative of three independent experiments. *C*, TAZ^4SA^-transformed S1 cells were infected with a lentivirus expressing shRNAs that target TEAD 1/3/4 and CRISPR to target TEAD2. RNA was isolated and qPCR was performed to quantify the expression of *TEAD*s1-4 and *LAMC2*, *LAMA3*, and *LAMB3* mRNAs. Three technical replicates are shown. ∗*p*-value < 0.05. Statistical significance was determined by two-sided, unpaired *t* test.

We analyzed our previously published RNA-seq data on constitutively active TAZ (TAZ^4SA^)-transformed mammary epithelial cells (12) for genes relating to ECM remodeling and differentiation, which revealed that TAZ represses the expression of all subunits of LM332 (LAMA3, LAMB3, and LAMC2), as well as the β4 integrin, consistent with their de-differentiated morphology (Figs. 1*A*and S1A). Given that the ability of TAZ to repress LM332 is novel, we pursued the mechanism involved. For this purpose, we focused on the LAMC2 subunit because it is unique to LM332. We validated the RNA-seq data by immunoblotting to confirm the downregulation of LAMC2 protein (Fig. 1*B*). We also confirmed that the ability of TAZ to repress LAMC2 is dependent on the TAZ DNA-binding partner TEAD because downregulation of TEAD1/2/3/4 significantly increased LAMC2 expression (Fig. 1*C*). Consistent with our RNA-seq data, we also found TEAD-dependent expression of other components of LM332: LAMA3 and LAMB3 upon TEAD downregulation (Fig. 1*C*). TAZ over-expression in S1-4SA-TAZ cells was confirmed by quantitative PCR (qPCR) (Fig. S1A).

**Figure 2:**
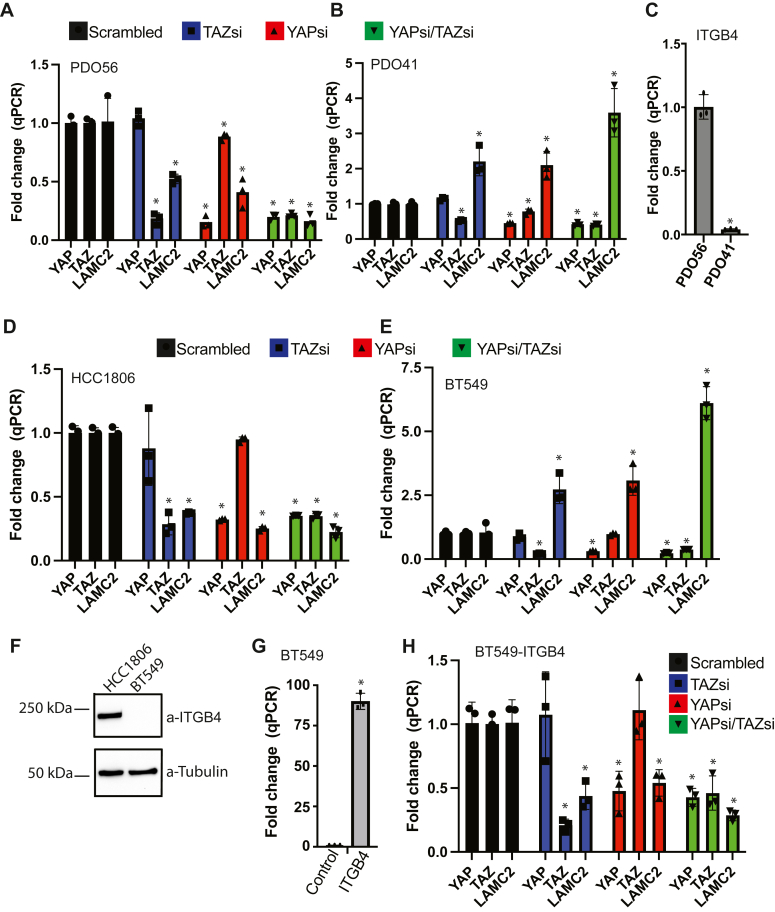
**Differential regulation of LAMC2 by YAP/TAZ.** Expression of either YAP, TAZ, or YAP and TAZ was downregulated using siRNAs in PDO56 (*A*), PDO41 (*B*), HCC1806 (*D*), and BT549 (*E*), and qPCR was performed on these transfected populations to quantify the expression of *YAP*, *TAZ*, and *LAMC2* mRNA. *C*, RNA was isolated from PDO56 and PDO41 PDOs, and qPCR was performed to quantify β4 integrin mRNA. *F*, cell lysates from HCC1806 and BT549 cells were immunoblotted using β4 and tubulin Abs. The immunoblot shown is representative of two independent experiments. *G*, BT549 cells were stably transfected with either a control vector or a β4 integrin overexpression plasmid. Isolated mRNA from these cells was used to perform qPCR to detect changes in ITGB4 expression. *H*, BT549 cells stably expressing β4 integrin were transfected with siRNA targeting YAP or TAZ or both. Isolated mRNA from these cells was used to perform qPCR to detect changes in the levels of YAP, TAZ, and LAMC2. ∗*p*-value < 0.05. Three technical replicates from a representative experiment are shown. Statistical significance was determined by two-sided, unpaired *t* test.

We extended our initial observations on TAZ-mediated repression of LAMC2 to two different organoids (patient-derived organoids, PDO41 and PDO56) that we established from freshly harvested breast tumors. Specifically, we downregulated the expression of either YAP or TAZ using siRNAs and assessed the impact on LAMC2 expression. Unexpectedly, the results differed between the two organoids: LAMC2 expression decreased in PDO56 (Fig. 2*A*) but increased in PDO41 (Fig. 2*B*). Similar opposing results were obtained in two breast cancer cell lines (HCC1806 and BT549). Diminishing either YAP or TAZ expression decreased LAMC2 in HCC1806 cells (Fig. 2*D*) but increased it in BT549 cells (Fig. 2*E*). One distinguishing feature of these cell lines is expression of the β4 integrin. We found that the expression of β4 is significantly lower in the PDO41 organoid than in the PDO56 (Fig. 2*C*). Expression of β4 is also markedly lower in BT549 cells, which have a mesenchymal morphology, than in HCC1806 cells, which have an epithelial morphology (Fig. 2*F*). These data suggested that the regulation of LAMC2 by YAP/TAZ is influenced by expression of the β4 integrin. To investigate a causal role for β4 in this regulation, we exogenously expressed this integrin subunit in BT549 cells (Fig. 2*G*) and observed that downregulating the expression of either YAP or TAZ decreased LAMC2 expression (Fig. 2*H*), indicating that the expression of β4 induces YAP/TAZ-mediated LAMC2 expression in these cells.

### The β4 integrin influences YAP/TAZ-mediated transcription of LAMC2 by regulating ZEB expression

**Figure 3:**
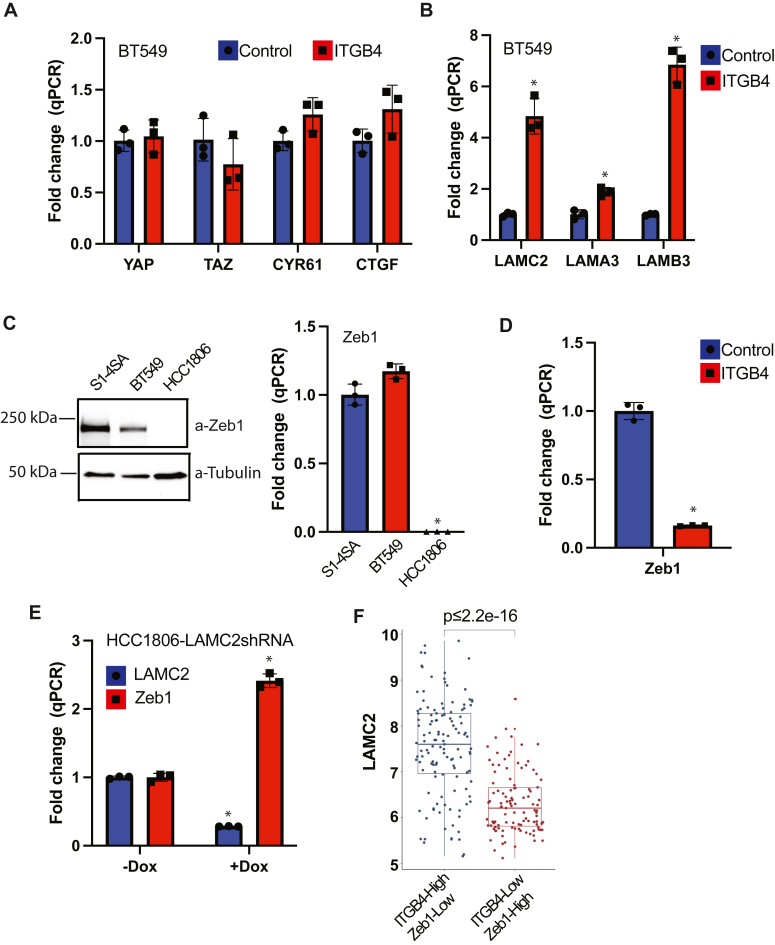
**The β4 integrin influences YAP/TAZ-mediated transcription of LAMC2.***A*, expression of the mRNAs for YAP, TAZ, and their target genes CYR61 and CTGF was compared between control and β4-expressing BT549 cells by qPCR. *B*, expression of LAMC2, LAMA3, and LAMB3 mRNA was compared between control and integrin β4-expressing BT549 cells by qPCR. *C*, *left panel*, cell lysates from S1-4SA, HCC1806, and BT549 cells were immunoblotted using ZEB1 and tubulin Abs. The immunoblot shown is representative of three independent experiments. *Right panel* shows mRNA changes in ZEB1 among these three cell lines. *D*, mRNA was collected from BT549 cells that express either vector control or β4, and qPCR was performed to quantitate changes in ZEB1 expression. *E*, HCC1806 cells stably expressing dox-inducible LAMC2 shRNA were incubated with or without Dox (1 ug/ml) for 48 h. Levels of LAMC2 and Zeb1 mRNA were quantified using qPCR. *F*, LAMC2 mRNA levels from the METABRIC dataset (15) were compared in β4 high/ZEB1 low and β4 low/ZEB1 high tumors. Expression values represent expression log intensity levels from an Illumina v3 microarray. ∗*p*-value < 0.05. Three technical replicates from a representative experiment are shown. Statistical significance was determined by two-sided, unpaired *t* test.

Surprisingly, we observed that exogenous expression of β4 in BT549 cells did not affect either the expression of YAP/TAZ or their activation as assessed by expression of their target genes (*CYR61, CTGF*) (Fig. 3*A*), but it did increase expression of all three subunits of LN332 (Fig. 3*B*). Based on this observation, we hypothesized that the β4 integrin affects a co-regulator of YAP/TAZ. We focused on the possible involvement of ZEB1 for several reasons. ZEB1 binds to the *LAMC2* promoter and represses its expression in prostate cancer cells (14). Also, ZEB1 is expressed in TAZ-transformed mammary cells and BT549 cells but not in HCC1806 cells (Fig. 3*C*). More importantly, we found that expression of β4 in BT549 cells decreased ZEB1 expression (Fig. 3*D*), suggesting a potential negative feedback loop between β4 and ZEB1. In fact, downregulation of ZEB1 by β4 requires its ligand LM332 based on our finding that downregulation of LAMC2 using a dox-inducible shRNA increased ZEB1 expression (Fig. 3*E*). To validate the relationship among β4, ZEB1, and LAMC2 in human tumors, we compared their mRNA levels in breast tumors (n = 1980) using data available from the METABRIC consortium (15). Tumors were classified as either β4^high^/ZEB1^low^ (tumors within the upper 20% of β4 expression and the lower 20% of ZEB1 expression, n = 127) or β4^low^/ZEB1^high^ (tumors within the lower 20% of β4 expression and the upper 20% of ZEB1 expression, n = 107). We found significant enrichment of LAMC2 in the β4^high^/ZEB1^low^ compared to the β4^low^/ZEB1^high^ group (Fig. 3*F*).

**Figure 4:**
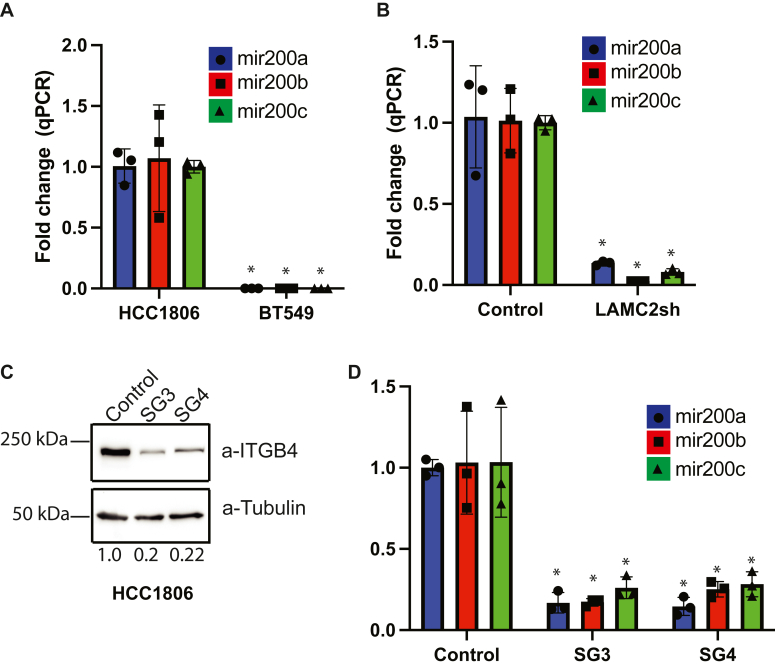
**The β4 integrin sustain expression of mir200s that targets ZEB1.***A*, expression of mir200a, mir200b, and mir200c was compared between BT549 and HCC1806 cells by qPCR. *B*, effect of LAMC2 downregulation on mir200a, mir200b, and mir200c in HCC1806 cells was quantified by qPCR. *C*, CRISPR-mediated downregulation of β4 integrin in HCC1806 was validated by immunoblotting using β4 and tubulin Abs. *D*, effect of β4 integrin downregulation on mir200a, mir200b, and mir200c expression in HCC1806 cells was quantified by qPCR. ∗*p*-value < 0.05. Three technical replicates from a representative experiment are shown. Statistical significance was determined by two-sided, unpaired *t* test.

To investigate the relationship between β4 and ZEB1 further, we evaluated the role of the mir-200s because they are known to repress ZEB1 (16) and our previous work highlighted the ability of β4 to regulate miR expression (17). Expression of mir200 is very low in BT549 compared to HCC1806 cells (Fig. 4*A*), which is consistent with their relative expression of ZEB1 (Fig. 3*C*). Also, downregulation of LAMC2 induced ZEB1 but reduced mir200 expression (Fig. 4*B*). To implicate a causal role for the β4 integrin in the regulation of mir-200, we knocked-out β4 expression in HCC1806 cells using CRISPR (Fig. 4*C*) and observed a significant decrease in mir-200 (Fig. 4*D*) as well as an increase in ZEB1 (Fig. S1B). Furthermore, reducing mir200 levels using an anti-miR in HCC1806 cells increased expression of Zeb1 (Fig. S1C). We did not observe any significant change in cell morphology upon our various manipulations of ITGB4 or LAMC2 (Fig. S2). These data indicate that the LM332–β4 axis suppresses ZEB1.

### ZEB1 interferes with TEAD binding to the LAMC2 promoter region

**Figure 5:**
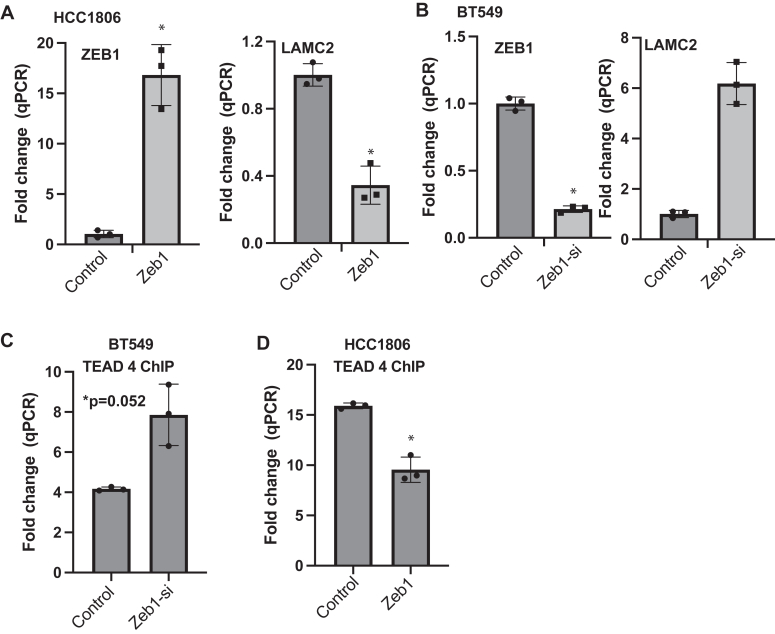
**ZEB1 interferes with TEAD binding to the LAMC2 promoter region.***A*, expression of ZEB1 and LAMC2 mRNAs in HCC1806 cells that had been transfected with either a control or a ZEB1 vector was quantified by qPCR. *B*, ZEB1 expression in BT549 cells was diminished using either control or ZEB1 siRNAs, and the effect on ZEB1 and LAMC2 mRNA expression were quantified by qPCR. *C*, ZEB1 expression in BT549 cells was diminished using either control or ZEB1 siRNAs. ChIP was performed using a TEAD antibody and primers targeting the promoter region of LAMC2 were used to amplify the precipitated DNA by qPCR. *D*, ChIP was performed on HCC1806 cells that had been transfected with either a control or a ZEB1 vector using a TEAD antibody and primers targeting the promoter region of LAMC2 were to amplify the precipitated DNA by qPCR. ∗*p*-value < 0.05 except for 5C, which is <0.52. Three technical replicates from a representative experiment are shown. Statistical significance was determined by two-sided, unpaired *t* test. ChIP, chromatin immunoprecipitation.

To understand the role of ZEB1 in the regulation of LAMC2, we expressed ZEB1 in HCC1806 cells and observed a significant decrease in LAMC2 expression (Fig. 5*A*). Conversely, diminishing ZEB1 expression in BT549 cells using siRNAs increased LAMC2 expression (Fig. 5*B*). Together, these results suggest that ZEB1 represses LAMC2 transcription mediated by YAP/TAZ/TEAD. To assess this possibility, we assayed TEAD binding to the *LAMC2* promoter region as a function of ZEB1 using chromatin immunoprecipitation (ChIP). Downregulation of ZEB1 expression in BT549 cells increased TEAD binding to the promoter (Fig. 5*C*). In contrast, exogenous expression of ZEB1 in HCC1806 cells decreased TEAD binding to the promoter (Fig. 5*D*). These findings indicate that ZEB1 represses *LAMC2* transcription by interfering with the ability of TEAD to bind to the *LAMC2* promoter. Given that ZEB1 is canonically a transcriptional repressor and histone deacetylase, it seems likely that it removes histone acetyl groups from the *LAMC2* promoter, condenses chromatin around this area, and blocks accessibility of this promoter to transcriptional activation by TAZ/TEAD.

### The LM332–β4 axis promotes ferroptosis resistance

**Figure 6:**
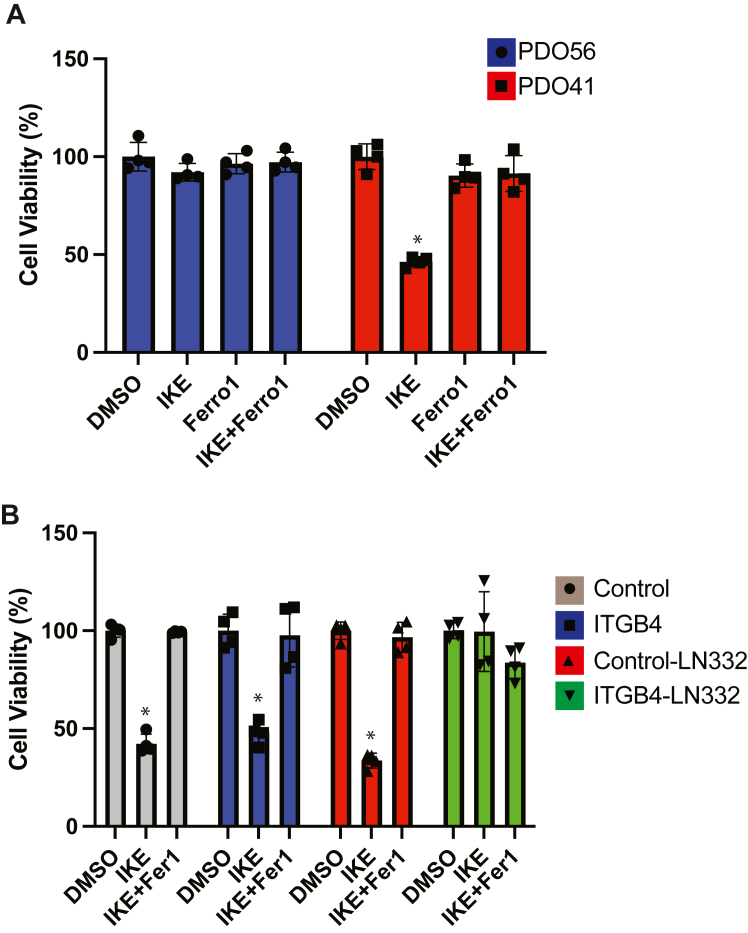
**The LM332–β4 axis promotes ferroptosis resistance.***A*, PDO56 and PDO41 PDOs were incubated with IKE (10 μM) or ferrostatin-1 (2 μM) for 48 h. Cell viability was quantified using Cell Titer-Glo. *B*, the PDO41 organoid was transfected with either a control or β4 integrin expression vector and then treated with either DMSO, IKE (10 μM), or IKE and ferrostatin-1 (2 μM) either in the presence or absence of LM332 (1 μg/ml) for 12 h. Cell viability was quantified using Cell Titer-Glo. ∗*p*-value < 0.05. Results from three independent organoid domes are shown. Statistical significance was determined by two-sided, unpaired *t* test. IKE, Imidazole Ketone Erastin; LM332, laminin 332.

Previously, we reported that the β4 integrin promotes resistance to ferroptosis in mammary epithelial cells and in carcinoma cells that express this integrin (6). This finding provided a mechanism for the observation that differentiated carcinoma cells are more ferroptosis resistant than cells with a mesenchymal phenotype (18). In the present study, we extended this observation to patient-derived organoids. The PDO56 organoid, which expresses relatively high levels of β4 (Fig. 2*C*), is resistant to imidazole ketone erastin (IKE), a drug that induces ferroptosis by inhibiting the xCT transporter (Fig. 6*A*). In contrast, the PDO41 organoid, which expresses low β4 (Fig. 2*C*), is sensitive to IKE, and this IKE-mediated cell death is rescued by the ferroptosis inhibitor ferrostatin-1 (Fig. 6*A*). The contribution of ECM to ferroptosis, however, has not been investigated. To address this issue, we expressed β4 in the PDO41 organoid and incubated these transfected organoids in the presence or absence of LM332. The induction of ferroptosis was evaluated in response to IKE. Ferroptosis resistance was observed only in the combined presence of β4 integrin and LM332 (Fig. 6*B*). These data demonstrate that the β4 integrin requires its matrix ligand to promote ferroptosis resistance. They also highlight the physiological significance of the ability of β4 to promote LM332 expression by repressing ZEB1 and enabling TEAD-mediated transcription of LAMC2.

## Discussion

This study provides evidence for a unique function of the β4 integrin in influencing the nature of YAP/TAZ/TEAD-mediated transcription that derives from its ability to repress ZEB1. Specifically, we demonstrate that YAP and TAZ can promote TEAD-mediated transcription of *LAMC2* in the presence of β4 because this integrin sustains the expression of mir-200s that target ZEB1. In the absence of β4, ZEB1 is expressed, and it disrupts the ability of TEAD to bind to the *LAMC2* promoter. These results build upon a previous study on prostate cancer cells that demonstrated that ZEB1 binds to the *LAMC2* promoter and represses its expression (14). This study, however, did not provide a mechanism for how ZEB1 is regulated nor did it implicate YAP/TAZ in the regulation of LAMC2. Our data also highlight the importance of the interaction of the ECM with integrins in repressing ZEB1 as evidenced by LM332/β4.

Our finding that YAP and TAZ can drive TEAD-mediated transcription in carcinoma cells with an epithelial phenotype has novelty, especially considering previous work demonstrating that TAZ expression and activation are elevated in poorly differentiated carcinomas and CSCs (2). Although TAZ expression and activation may be lower in epithelial cells, our data indicate that YAP and TAZ can be active in these cells to facilitate the TEAD-mediated transcription of other epithelial genes as exemplified by *LAMC2*. Our results also provide potential insight into why YAP and TAZ do not promote de-differentiation in epithelial cells in the presence of β4 because this integrin represses ZEB1, a master regulator of EMT and stemness (19). More specifically, we suggest that the expression of miR200s, which is sustained by β4, can inhibit the ability of YAP/TAZ to induce an EMT unless the level of YAP/TAZ activation is such that it can overwhelm the impact of mir200s as observed with constitutively active TAZ (TAZ^4SA^) (Fig. 1). The transcriptional regulation of ZEB1 by TAZ^4SA^ could explain why TAZ is an inhibitor of LAMC2 in mesenchymal contexts.

A significant conclusion from our study is that LM332 promotes ferroptosis resistance in the presence of the β4 integrin. Although there has been a plethora of studies on ferroptosis resistance mechanisms, the contribution of the ECM has not been a focus. A role for LM332 in ferroptosis resistance is consistent with reports that cells with epithelial differentiation are more resistant to drugs that induce ferroptosis compared to mesenchymal cells (18). This finding also adds to our previous work implicating β4 in ferroptosis resistance (6). It is now apparent, however, that β4 must engage its matrix ligand to promote this resistance (see Fig. 6*B*).

## Experimental procedures

### Reagents

IKE was purchased from Med Chem Express (HY-114481). Ferrostatin-1 was purchased from Sigma (SML0583). TRIzol reagent (15596026) was purchased from Thermo fisher scientific. Silencer Select siRNA to Zeb1 (assay id s229971) was purchased from Ambion. Dharmafect4 reagent was purchased from Dharmacon. The siRNA targeting TAZ and YAP was synthesized by Dharmacon and sequence was described previously (20). LM332 was purchased from Biolamina. We purchased mirVana miRNA inhibitor and hsa-miR-200 (a, b, and c) from Invitrogen.

### Cell lines and patients-derived organoids

BT549 and HCC1806 were kindly provided by Dr Dohoon Kim (UMass Chan Medical School). HMT-3522 S1 cells were obtained from Dr. Mina Bissell (Lawrence Berkeley National Laboratory) and transfected with a constitutively activate TAZ construct (TAZ^4SA^) (provided by Dr Xaralabos Varelas, Boston University). These cells are referred as 4SA-TAZ. The PDOs were established from tumor tissues obtained from UMass Cancer center tumor bank as described (21). BT-549 cells expressing the β4 integrin were generated by transfecting a β4 plasmid (purchased from Vector Builder). The cells were FACS-sorted using a β4 integrin antibody (439-9b, eBioscience). HCC-1806 expressing an inducible LAMC2 shRNA (Inducible Human LAMC2 shRNA from Horizon Discovery) were generated and LAMC2 was downregulated by incubating these cells in doxycycline (1 μg/ml) for 48 h. A ZEB1 expression plasmid was purchased from Vector Builder and stably transfected into HCC-1806 cells. Two guide RNAs targeting the β4 integrin were used to downregulate β4 integrin expression in HCC1806 cells as described previously (6, 22). The plasmid expressing shTEAD1/3/4 and TEAD2 CRISPR were kindly provided by Dr Junhao Mao and described previously (23). Freshly collected tumor tissues were partially digested and cultured as organoids by embedding them in reduced growth factor basement membrane extract (Cultrex). The embedded organoids were cultured using organoid media, whose composition was described before (24). PDO-56 was derived from a human specimens diagnosed as an invasive lobular carcinoma, with no identified lymphovascular invasion. This specimen was positive for ER but negative for PR and HER2. PDO-41 was derived from a human specimen diagnosed as a ductal carcinoma *in situ* and negative for ER, PR, and HER2.

### Chromatin immunoprecipitation

ChIP was performed with the ChIP-IT Express Kit (Active Motif) using a TEAD4 Ab that was purchased from Santa Cruz Biotechnology (N-G2). The primers used for qPCR of the *LAMC2* promoter region, which were described previously (14), are as follows: FW: CGGTTTGTGCTCTGTGTGTT; RW: GAGCCTGTGTTTCAGGGTGT.

### qPCR

RNA was isolated using Nucleospin RNA kit (Macherey-Nagel) and cDNAs were produced using the Azura cDNA synthesis kit (Azura Genomics). The qPCR was performed using Azura View GreenFast qPCR mix (Azura Genomics). The results presented were normalized to GAPDH. The primer sequences were obtained from the primer bank (https://pga.mgh.harvard.edu/primerbank/).

### microRNA quantification

Total RNA was isolated from cells using Trizol following the manufacturer’s instructions. The isolated RNA was converted to cDNA using TaqMan Advanced miRNA cDNA Synthesis Kit (Applied Biosystem). The following Taqman assays were purchased from Thermo Fisher Scientific: hsa-let-7a-5p (478575_mir), hsa-miR-200a-3p (478490_mir), hsa-miR-200b-3p (477963_mir), and hsa-miR-200c-3p (478351_mir) and qPCR was performed using Taqman Fast Advanced Master Mix (4444557).

### Immunoblotting

Cells were lysed using RIPA buffer. Proteins were separated by SDS-PAGE and immunoblotted using either a β4 (EPR8558, AbCaM), Zeb1 (Cell Signaling), LAMC2 (SantaCruz SC-28330), or tubulin (kindly provided by Dr Leslie Shaw) Abs. Densitometric analysis of the immunoblots was done using the Gel Analyzer tool in Fiji. For each immunoblot, we measured the area under the peak and normalized it to the area under the peak with the corresponding loading control.

### Cell viability

PDOs plated in 24-well plates were treated with IKE and ferrostatin-1 as described in the legend to Figure 6. After incubation, the Cell Titer-Glo 3D reagent (Promega) (100 μl) was added to each well and incubated for 30 min. The contents of each well were transferred into black-wall 96-well plates (Corning), and luminescence was measured using a GloMax plate reader (Promega).

### RNA sequencing

The RNA sequencing data shown in Figure 1*A* was generated in a previously published study (25). The dataset is available in GEO (GSE115057).

### Statistics

Statistical significance for all figures was calculated using Student *t* test using Graphpad Prism 9.0. Sample normal distribution is confirmed with Prism using the Shapiro-Wilk (W) test.

## Data availabilty

All supporting data are contained within the manuscript.

## Supporting information

This article contains supporting information.

## Conflict of interest

The authors declare that they have no conflicts of interest with the contents of this article.

## References

[1] Piccolo S., Panciera T., Contessotto P., Cordenonsi M. (2023). YAP/TAZ as master regulators in cancer: modulation, function and therapeutic approaches. Nat. Cancer.

[2] Cordenonsi M., Zanconato F., Azzolin L., Forcato M., Rosato A., Frasson C. (2011). The Hippo transducer TAZ confers cancer stem cell-related traits on breast cancer cells. Cell.

[3] Lee E.C., Lotz M.M., Steele G.D., Mercurio A.M. (1992). The integrin alpha 6 beta 4 is a laminin receptor. J. Cell Biol..

[4] Shaw L.M., Rabinovitz I., Wang H.H., Toker A., Mercurio A.M. (1997). Activation of phosphoinositide 3-OH kinase by the alpha6beta4 integrin promotes carcinoma invasion. Cell.

[5] Chang C., Goel H.L., Gao H., Pursell B., Shultz L.D., Greiner D.L. (2015). A laminin 511 matrix is regulated by TAZ and functions as the ligand for the alpha6Bbeta1 integrin to sustain breast cancer stem cells. Genes Dev..

[6] Brown C.W., Amante J.J., Goel H.L., Mercurio A.M. (2017). The alpha6beta4 integrin promotes resistance to ferroptosis. J. Cell Biol..

[7] Kajiji S., Tamura R.N., Quaranta V. (1989). A novel integrin (alpha E beta 4) from human epithelial cells suggests a fourth family of integrin adhesion receptors. EMBO J..

[8] Hemler M.E., Crouse C., Sonnenberg A. (1989). Association of the VLA alpha 6 subunit with a novel protein. A possible alternative to the common VLA beta 1 subunit on certain cell lines. J. Biol. Chem..

[9] Englund J.I., Bui H., Dinc D.D., Paavolainen O., McKenna T., Laitinen S. (2022). Laminin matrix adhesion regulates basal mammary epithelial cell identity. J. Cell Sci..

[10] Lu S., Simin K., Khan A., Mercurio A.M. (2008). Analysis of integrin beta4 expression in human breast cancer: association with basal-like tumors and prognostic significance. Clin. Cancer Res..

[11] Chang C., Yang X., Pursell B., Mercurio A.M. (2013). Id2 complexes with the SNAG domain of Snai1 inhibiting Snai1-mediated repression of integrin beta4. Mol. Cell Biol..

[12] Geng Y., Amante J.J., Goel H.L., Zhang X., Walker M.R., Luther D.C. (2020). Differentiation of cancer stem cells through nanoparticle surface engineering. ACS Nano.

[13] Miner J.H., Yurchenco P.D. (2004). Laminin functions in tissue morphogenesis. Annu. Rev. Cell Dev. Biol..

[14] Drake J.M., Barnes J.M., Madsen J.M., Domann F.E., Stipp C.S., Henry M.D. (2010). ZEB1 coordinately regulates laminin-332 and beta4 integrin expression altering the invasive phenotype of prostate cancer cells. J. Biol. Chem..

[15] Curtis C., Shah S.P., Chin S.F., Turashvili G., Rueda O.M., Dunning M.J. (2012). The genomic and transcriptomic architecture of 2,000 breast tumours reveals novel subgroups. Nature.

[16] Perdigao-Henriques R., Petrocca F., Altschuler G., Thomas M.P., Le M.T., Tan S.M. (2016). miR-200 promotes the mesenchymal to epithelial transition by suppressing multiple members of the Zeb2 and Snail1 transcriptional repressor complexes. Oncogene.

[17] Gerson K.D., Maddula V.S., Seligmann B.E., Shearstone J.R., Khan A., Mercurio A.M. (2012). Effects of beta4 integrin expression on microRNA patterns in breast cancer. Biol. Open.

[18] Viswanathan V.S., Ryan M.J., Dhruv H.D., Gill S., Eichhoff O.M., Seashore-Ludlow B. (2017). Dependency of a therapy-resistant state of cancer cells on a lipid peroxidase pathway. Nature.

[19] Kroger C., Afeyan A., Mraz J., Eaton E.N., Reinhardt F., Khodor Y.L. (2019). Acquisition of a hybrid E/M state is essential for tumorigenicity of basal breast cancer cells. Proc. Natl. Acad. Sci. U. S. A..

[20] Dupont S., Morsut L., Aragona M., Enzo E., Giulitti S., Cordenonsi M. (2011). Role of YAP/TAZ in mechanotransduction. Nature.

[21] Xu Z., Goel H.L., Burkart C., Burman L., Chong Y.E., Barber A.G. (2023). Inhibition of VEGF binding to neuropilin-2 enhances chemosensitivity and inhibits metastasis in triple-negative breast cancer. Sci. Transl Med..

[22] Brown C.W., Amante J.J., Mercurio A.M. (2018). Cell clustering mediated by the adhesion protein PVRL4 is necessary for alpha6beta4 integrin-promoted ferroptosis resistance in matrix-detached cells. J. Biol. Chem..

[23] Liu X., Li H., Rajurkar M., Li Q., Cotton J.L., Ou J. (2016). Tead and AP1 coordinate transcription and motility. Cell Rep..

[24] Sachs N., de Ligt J., Kopper O., Gogola E., Bounova G., Weeber F. (2018). A living biobank of breast cancer organoids captures disease heterogeneity. Cell.

[25] Mukhopadhyay D., Goel H.L., Xiong C., Goel S., Kumar A., Li R. (2023). The calcium channel TRPC6 promotes chemotherapy-induced persistence by regulating integrin alpha6 mRNA splicing. Cell Rep..

