# Supplementary material for: YAP/TAZ-mediated regulation of laminin 332 is enabled by β4 integrin repression of ZEB1 to promote ferroptosis resistance
Source: J Biol Chem. 2024 Mar 18;300(4):107202. doi: 10.1016/j.jbc.2024.107202 (PMC11017052; doi:10.1016/j.jbc.2024.107202)
Supplement: Supplemental Figure S1 [file mmc1.pdf]

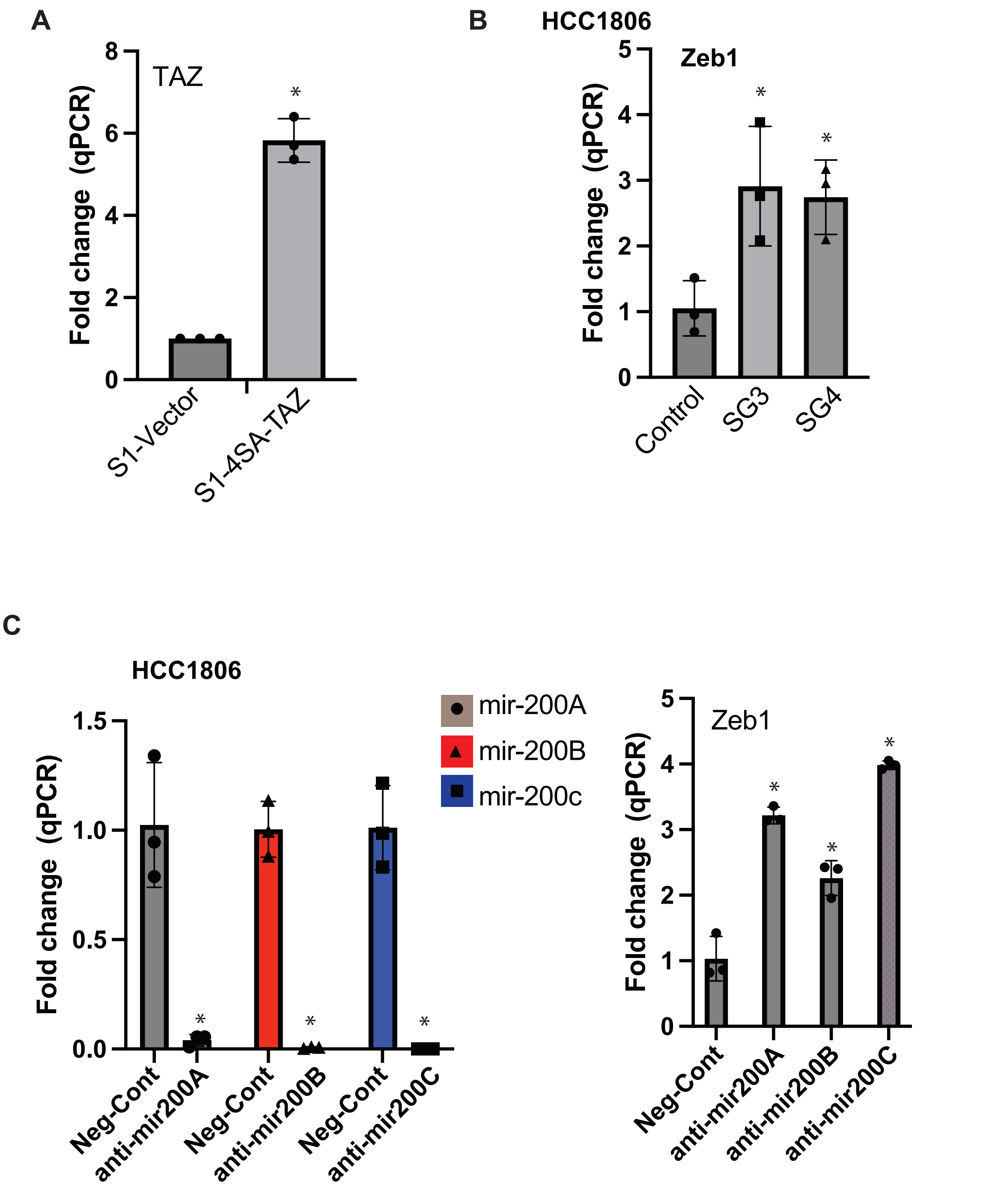

Figure S1. A. TAZ mRNA from control and TAZ4SA transformed S1 mammary epithelial cells was quantified by qPCR. Three biological replicates are presented. B. Effect of 4 integrin downregulation on ZEB1 expression in HCC1806 cells was quantified by qPCR. C. Left panel: Effect of mir200s antimirs on mir200s expression in HCC1806 cells was quantified by qPCR. Right panel: Effect of mir200 downregulation using antimirs on ZEB1 expression in HCC1806 cells was quantified by qPCR. * p-value <0.05. Three technical replicates from a representative experiment is shown. Statistical significance was determined by two-sided, unpaired t test.
