# Supplementary material for: YAP/TAZ-mediated regulation of laminin 332 is enabled by β4 integrin repression of ZEB1 to promote ferroptosis resistance
Source: J Biol Chem. 2024 Mar 18;300(4):107202. doi: 10.1016/j.jbc.2024.107202 (PMC11017052; doi:10.1016/j.jbc.2024.107202)
Supplement: Supplemental Figure S2 [file mmc2.pdf]

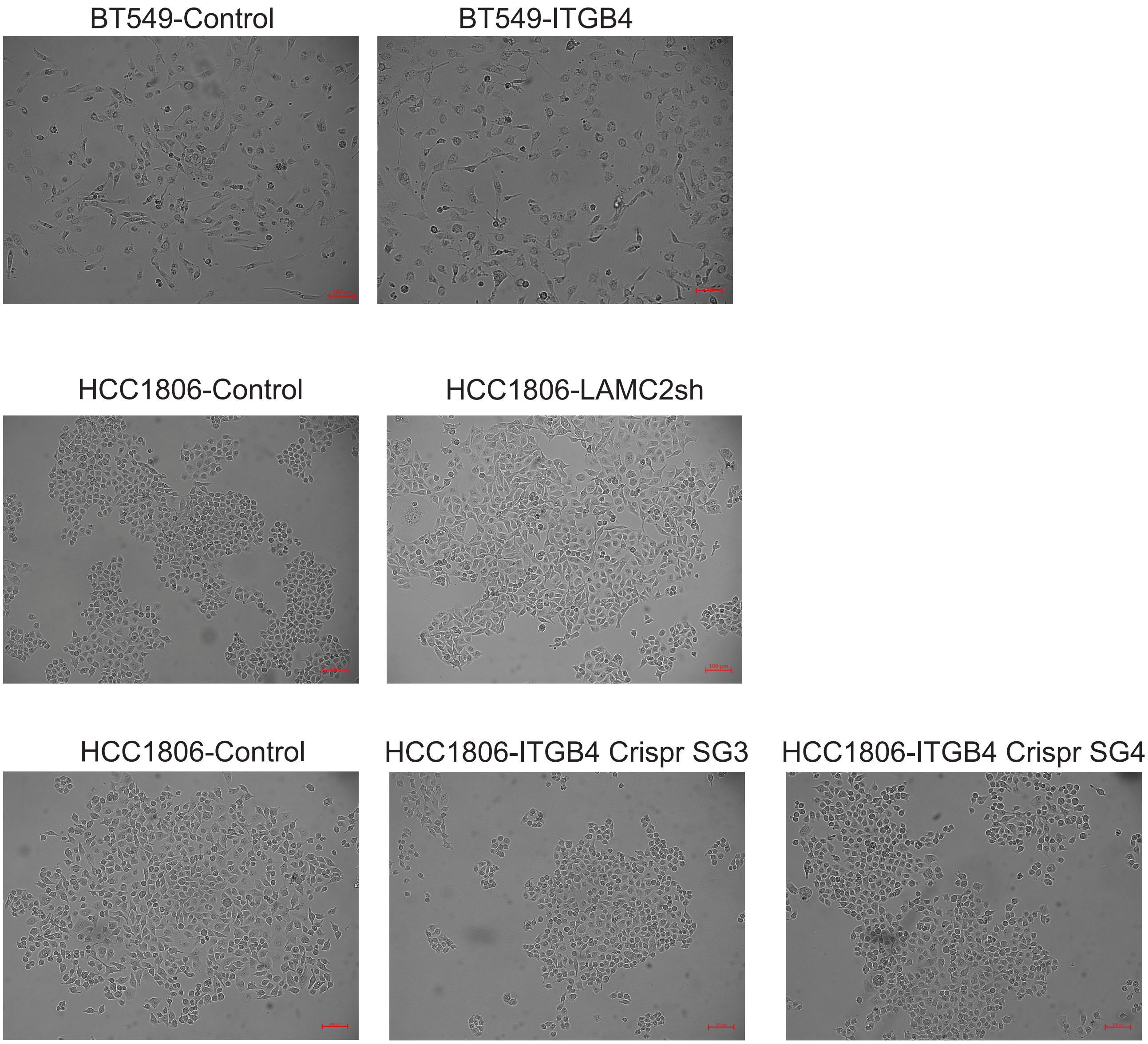

Figure S2. Bright field images of HCC1806 and BT549 cells in which the expression of either LAMC2 or ITGB4 was deleted using CRISPR. Scale bar = 100uM.
